# Human tumor suppressor PDCD4 directly interacts with ribosomes to repress translation

**DOI:** 10.1038/s41422-024-00962-z

**Published:** 2024-04-19

**Authors:** Xianwen Ye, Zixuan Huang, Yi Li, Mengjiao Wang, Wanyu Meng, Maojian Miao, Jingdong Cheng

**Affiliations:** Minhang Hospital & Institutes of Biomedical Sciences, Shanghai Key Laboratory of Medical Epigenetics, International Co-laboratory of Medical Epigenetics and Metabolism, Fudan University, Shanghai, China

**Keywords:** Ribosome, Cryoelectron microscopy

Dear Editor,

Protein translation regulation is a crucial and tightly controlled regulatory process that contributes to phenotypic diversity among cells with identical or similar genotypes. Across all the steps of translation, initiation is the most energy- and time-intensive stage. In eukaryotes, this process starts with the assembly of the 43S preinitiation complex (PIC), comprising 40S ribosome, eIF1, eIF1A, the eIF3 complex (eIF3A-M), eIF5, and the ternary complex (TC, consisting of eIF2α/β/γ, tRNA^iMet^, and GTP). After 43S PIC assembly, the eIF4F complex (consisting of the DEAD box helicase eIF4A, eIF4B, eIF4E, and eIF4G) is recruited, along with the mRNA, to form the 48S initiation complex (IC). Following the recognition of the first cognate AUG start codon by the 48S IC, the 60S ribosome joins to initiate translation elongation. This process requires a coordination of multiple complexes and factors for rigorous regulation of protein translation.^1^ Importantly, cells employ various mechanisms to inhibit translation initiation in response to environmental stress conditions, yet the detailed molecular mechanisms have not been fully elucidated.

PDCD4 functions as a translational repressor by interacting with the initiation factor eIF4A through the MA3 domains (Fig. 1a), thereby preventing its incorporation into the eIF4F complex.^2,3^ Besides this crucial role, its mechanism of action is currently poorly understood. To investigate the role of PDCD4 in translation regulation, wild-type PDCD4 with a C-terminal GFP tag was expressed in PDCD4 knockout human DLD-1 cells. Consistent with previous reports,^4^ PDCD4 predominantly resides in the nucleoplasm under normal growth conditions. However, stress exposure such as DNA damage or nutrient starvation induced the translocation of PDCD4 into the cytoplasm (Supplementary information, Fig. S1). Subsequently, we fractionated cell lysates from wild-type human HEK293T cells under glucose starvation conditions (treated for 24 h) using a 10%–40% sucrose gradient, enabling the analysis of endogenous PDCD4 distribution among different ribosomal populations. Compared to PDCD4 in the nucleus of normally growing cells, PDCD4 mainly (> 10-fold enriched) associated with the 40S ribosome peak in the cytosol under the starvation condition (Fig. 1b). We did notice a small amount of PDCD4 in the cytoplasm of the control cells. While we cannot entirely dismiss the possibility that a small amount of PDCD4 might be associated with the 40S ribosome in the cytosol, it is plausible that this association results from the rapid export of PDCD4 triggered by the stress conditions during cell preparation for lysis. Supporting this hypothesis, we observed a rapid export of PDCD4 during the incubation with buffer solutions (Supplementary information, Fig. S1b).

**Fig. 1:**
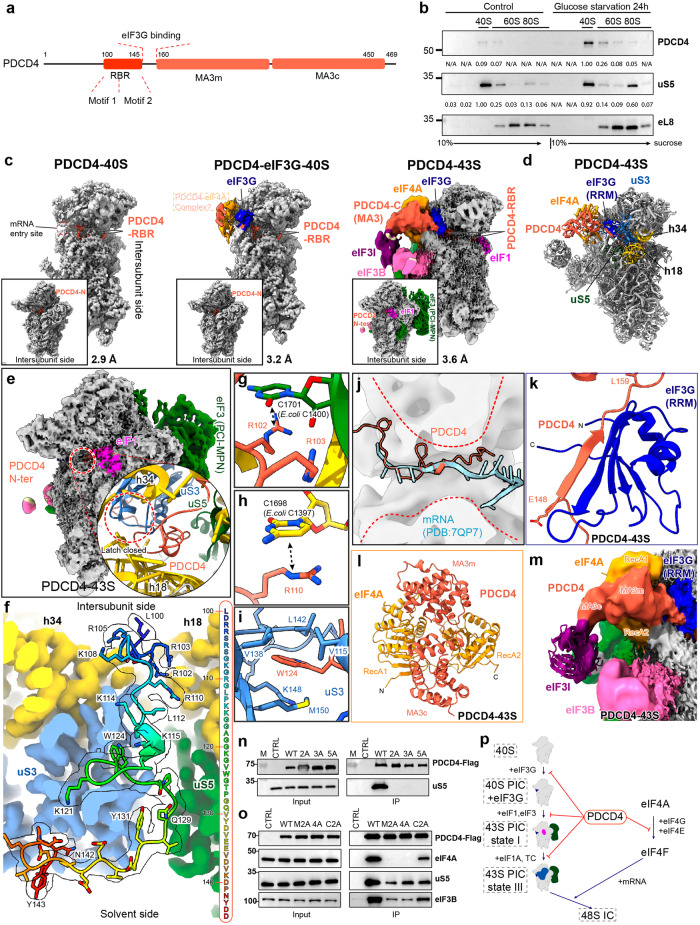
Structural and biochemical analyses of the PDCD4–ribosome complex. **a** Schematic showing the domain architecture of the human PDCD4 protein. **b** Human HEK293T cells were either subjected to 24 h of glucose starvation or left untreated. Subsequently, the cytoplasmic lysates were subjected to fractionation on a sucrose gradient ranging from 10% to 40%. PDCD4 antibody was used to detect the distribution of the endogenous PDCD4 over different ribosome populations. Intensity, normalized to a maximum of 1.00, was calculated using ImageJ software. **c** Cryo-EM maps of PDCD4–40S (left), PDCD4–eIF3G–40S (middle) and PDCD4–43S (right). The composite maps shown are derived from multi-body refinement and after local resolution filtering using either Relion (PDCD4–43S) or DeepEMhancer (PDCD4–40S and PDCD4–eIF3G–40S). The density of the PDCD4–eIF4A complex in the PDCD4–eIF3G–40S map is shown at the lower contour level. The label of the PDCD4–eIF4A complex is boxed out, indicating that it represents only a putative model. **d** Molecular model of the PDCD4–43S complex highlighting the positions of the PDCD4–eIF4A complex and eIF3G (blue). Helix 18 and helix 34 of the 18S rRNA are colored in yellow, while uS3 and uS5 are represented in blue and green, respectively. **e** Cryo-EM map of the PDCD4–43S state filtered according to its local resolution. A zoomed insert highlights the closed “latch” region (red circle) within the mRNA channel, in contrast to the open latch in the 43S PIC “State III”. **f** Overview of the interactions of PDCD4 RBR with the mRNA channel. The 40S is shown as a colored density map derived from the PDCD4–43S state, while the RBR model is shown as sticks fitted into density (transparent) and colored in rainbow. The complete sequence of the RBR is also shown on the right. **g**–**i** Detailed interactions between RBR and the 40S subunit: R102 stacks with base C1701, and R103 interacts with the 18S rRNA backbone (**g**); R110 stacks with base C1698 (**h**); W124 inserts into a hydrophobic pocket in uS3 (**i**). **j** PDCD4 spatially clashes with the mRNA in the 48S IC (PDB: 7QP7). The mRNA entry channel is indicated by red dashed lines. **k** A region (amino acids 148–159) of PDCD4 forms an antiparallel β-sheet with the RRM domain of eIF3G (blue). **l** The overall conformation of the PDCD4–eIF4A complex in the PDCD4–43S state. **m** Zoomed view highlighting the direct contact between MA3c of PDCD4 and eIF3I (purple). The fitted model for eIF3I is shown to better illustrate its position. **n**, **o** Co-IP experiments were performed in human HEK293T cells transiently transfected with PDCD4-Flag and its mutants using anti-Flag beads. Interactions between PDCD4-Flag and the 40S ribosome (**n**) or eIF4A (**o**) were detected by immunoblotting for uS5 and eIF4A, respectively. CTRL: Control. The PDCD4 protein was detected by immunoblotting for the Flag tag. **p** Proposed model for PDCD4-mediated inhibition of 43S PIC assembly.

We next pursued the structural investigation by using a tetracycline-inducible PDCD4-Flag protein as bait to purify its associated native complexes from densely cultured human HEK 293/Flp-In/T-Rex cells. Our ensemble single-particle cryo-EM analysis revealed three distinct structures, termed PDCD4–40S, PDCD4–eIF3G–40S, and PDCD4–43S (Fig. 1c). We were able to resolve the structures of PDCD4–40S and PDCD4–eIF3G–40S at resolutions of 2.9 Å and 3.2 Å, respectively, while the reconstruction of the PDCD4–43S remained a lower resolution (Fig. 1c; Supplementary information, Figs. S2–S4, Tables S1, S2 and Data S1). However, serendipitously, we obtained an identical PDCD4-containing 43S state at 3.6 Å resolution from the sample that was derived from cycloheximide (CHX)-treated (CHX was added to prevent ribosome runoff) HEK 293/Flp-In/T-Rex cells using the tetracycline-inducible PYM1-Flag as bait (Fig. 1c; Supplementary information, Figs. S3–S6, Tables S1, S2 and Data S1). PYM1 was believed to remove all associated exon junction complexes during the pioneer round of translation. However, for unknown reasons, PDCD4 was enriched in the PYM1 pull-out sample, as confirmed by the MS analysis (Supplementary information, Data S1).

In all three identified states, an N-terminal segment of PDCD4 (amino acids 100–145) is positioned within the mRNA entry channel (Fig. 1c). We thus refer to this segment as the ribosome-binding region (RBR, Fig. 1a). The PDCD4–40S state represents an idle 40S ribosomal subunit bound to PDCD4 via the RBR, while the rest of PDCD4 is invisible due to its flexibility (Fig. 1c; Supplementary information, Fig. S7). The structure of the PDCD4–eIF3G–40S state closely resembles that of PDCD4–40S state but with additional density for the initiation factor eIF3G adjacent to the mRNA entry site; this is the same position as previously observed in the 43S PIC (Fig. 1c; Supplementary information, Fig. S7).^5–8^ In the PDCD4–43S state, an extra structured and well-resolved density was observed, allowing the assignment of the C-terminal MA3 domains of PDCD4 and one copy of eIF4A (Fig. 1c, d; Supplementary information, Fig. S7). However, the corresponding density in the PDCD4–eIF3G–40S structure was less highly resolved, suggesting the notable flexibility in this region at this state (Fig. 1c; Supplementary information, Fig. S7).

In the PDCD4–43S state, the 43S PIC closely resembles the previously described intermediate “State I” of the 43S PIC assembly, characterized by the presence of the eIF1 and eIF3 complex but the absence of the TC and eIF1A (Fig. 1e; Supplementary information, Fig. S8a).^7^ Transitioning from “State I” to the fully assembled 43S PIC “State III” necessitates the recruitment of initiation factors eIF1A and TC, leading to the opening of the mRNA entry channel at the latch region (latch open) (Supplementary information, Fig. S8b).^7,9^ However, in PDCD4–43S, the latch remains closed (latch closed), as indicated by the proximity of uS3 and h18 (Fig. 1e). This closed arrangement is stabilized by the RBR of PDCD4 occupying the mRNA entry channel (Fig. 1e). Moreover, as revealed by 3D classification, PDCD4 was exclusively found in the early “State I” but not in the latter “State II” or “State III” states of the 43S PIC assembly (Supplementary information, Fig. S3). This finding suggests that PDCD4 plays a role in inhibiting the early phase of 43S PIC assembly.

Specifically, we observed that amino acids 100–145 of PDCD4 RBR are positioned within the 40S mRNA entry channel, extending from the decoding center (DC) on the intersubunit side through the channel toward the mRNA entry side (Fig. 1f). The RBR region can be divided into two segments: the upstream segment (amino acids 100–112), featuring a basic residue-rich “Motif 1”, and the second segment (amino acids 113–143), which includes “Motif 2”, characterized by a conserved “WG” dipeptide (Supplementary information, Fig. S9). Both segments exhibit intensive interactions with the mRNA channel wall formed by uS3, uS5, and the 18S rRNA (Fig. 1g–i; Supplementary information, Fig. S10a–f). Remarkably, PDCD4 RBR not only sterically blocks mRNA and initiator tRNA^iMet^ binding to prevent the formation of the 48S IC,^6,8^ but also coincides with the position of eIF1A in 43S/48S complexes (Fig. 1j; Supplementary information, Fig. S10g, h),^6–8^ explaining the absence of eIF1A in all our structures. Additionally, PDCD4 adopts a very similar conformation to the previously published ribosome hibernation factors SERBP1 and HABP4 (Supplementary information, Fig. S10i).^10^ These proteins not only bind to the same surface on the 40S ribosome but also share very high sequence similarity with PDCD4, especially at Motifs 1 and 2 (Supplementary information, Fig. S9). Moreover, PDCD4 shares binding sites with the general translation inhibitor NSP1 from SARS-CoV-2 (Supplementary information, Fig. S10j).^10^ These findings suggest that PDCD4 RBR occupies the mRNA entry channel and prevents further assembly of 43S PIC by likely hindering eIF1A and TC binding.

In addition to PDCD4 RBR, the PDCD4–43S revealed the positioning of the C-terminal MA3 domains of PDCD4 and one copy of eIF4A above the mRNA entry site. The main bridge between the 40S ribosome and the C-terminal MA3 domains is eIF3G. Stable contact between eIF3G and PDCD4 is established via a short stretch of PDCD4 (amino acids 148–159) adjacent to the RBR, which forms an antiparallel β-sheet with the RNA recognition motif (RRM) of eIF3G (Fig. 1k; Supplementary information, Fig. S5c–e). This positions the MA3 domains and eIF4A between the 40S head and the β-propeller domain of the initiation factor eIF3I. The confirmation of the PDCD4–eIF4A complex in the PDCD4–43S state is very similar to the crystal structure of the eIF4A–PDCD4 complex (Fig. 1l; Supplementary information, Fig. S11a). However, we observed that only one copy of eIF4A bound to both MA3 domains of PDCD4 (two-MA3 binding mode) (Supplementary information, Fig. S11a).^11,12^ The other copy that only binds to MA3c (MA3c binding mode) in the crystal structures is missing in the PDCD4–43S state (Fig. 1m). Instead, the MA3c domain directly contacts eIF3I, which is incompatible with eIF4A positioning in the crystal structures (Supplementary information, Fig. S11b).^11,12^ These results confirm that the “two-MA3 binding mode” in our PDCD4–43S structure is reflective of the physiological interaction mode of the PDCD4–eIF4A complex.

A study by Querido et al.^5^ observed two eIF4A molecules in a fully assembled 48S IC, one at the mRNA exit site^8^ and the other, similar to our findings, at the mRNA entry site. Although the second eIF4A is likely to be active for mRNA unwinding in this position, the eIF4A observed in our PDCD4–43S state is in an inhibited state (PDCD4 blocks the mRNA-binding interface of eIF4A) (Supplementary information, Fig. S11c), complexed with PDCD4 at the mRNA entry site (Fig. 1d). We thus suggest that this position at the mRNA entry site serves both as a recruitment hub and an inhibition platform for eIF4A.

Based on our structural analysis, we performed mutagenesis studies to validate our structural findings. This included the following mutations: “2A”, “3A” and “5A”, targeting “Motif 1/2”; Δ150–160, disrupting the eIF3G interaction; Δ100–160, removing the RBR and eIF3G interacting region; “M2A” and “4A”, impairing the “two-MA3 binding mode” interface;^12^ and “C2A”, impairing the “MA3c binding mode” interface^12^ (Supplementary information, Fig. S12a). Compared with the wild-type PDCD4, co-immunoprecipitation (Co-IP) experiments showed that the “2A”, “3A” and “5A” mutants completely lost their ability to bind to the ribosome, as indicated by immunoblotting of the ribosomal protein uS5 (Fig. 1n). The mutants “M2A” and “4A” completely lost eIF4A binding but retained the interaction with the initiation factor eIF3B and the ribosome, while “C2A” exhibited only reduced binding (Fig. 1o), confirming the “two-MA3 binding mode” hypothesis.^12^ Consistent with the Co-IP results, the “2A”, “3A”, “5A” and Δ100–160 mutations abolished their comigration with the 40S peak in the sucrose gradient assay (Supplementary information, Fig. S12b). Notably, the Δ150–160 mutation only showed a decreased association, suggesting that eIF3G plays a nonessential role (Supplementary information, Fig. S12b). However, none of the mutations in the MA3 domains (“M2A”, “C2A” and “4A”) affected its association with the 40S ribosome (Supplementary information, Fig. S12b). Collectively, these data underscore that both “Motif 1” and “Motif 2” in the RBR of PDCD4 are crucial for the association with the ribosome, irrespective of eIF4A interaction.

Based on our studies, we propose a model for the function of PDCD4 in inhibiting translation initiation (Fig. 1p): during initiation, the free idle 40S subunit (e.g., after successful recycling, phase 1) associates with eIF3G to form an intermediate (phase 2). Subsequently, eIF1 and the remaining components of the eIF3 complex are recruited to form the 43S “State I” (phase 3), and the final recruitment of eIF1A and TC leads to the canonical 43S “State III” (phase 4). Under stress, PDCD4 relocates to the cytoplasm and disrupts phases 1–3 by occupying the mRNA entry channel with its RBR and positioning eIF4A in its inhibited form via its MA3 domains (Fig. 1p). Thus, PDCD4 not only hampers the activity of eIF4A and the function of the eIF4F complex, but also directly inhibits the ribosome itself, independent of the PDCD4–eIF4A interaction. Our research establishes a link between tumorigenesis and the suppression of translation initiation, providing valuable insights into the underlying mechanisms of translation regulation.

### Supplementary information

Supplementary information, MATERIALS AND METHODS

Supplementary information, Fig. S1

Supplementary information, Fig. S2

Supplementary information, Fig. S3

Supplementary information, Fig. S4

Supplementary information, Fig. S5

Supplementary information, Fig. S6

Supplementary information, Fig. S7

Supplementary information, Fig. S8

Supplementary information, Fig. S9

Supplementary information, Fig. S10

Supplementary information, Fig. S11

Supplementary information, Fig. S12

Supplementary information, Table S1

Supplementary information, Table S2

Supplementary information, Data S1

## Data Availability

All cryo-EM maps and molecular models have been deposited in the Electron Microscopy Data Bank (EMDB) with accession IDs EMD-38752 (state PDCD4–40S), EMD-38753 (state PDCD4–eIF3G–40S), EMD-38754 (state PDCD4–43S), and in the Protein Data Bank (PDB) with accession codes 8XXL (state PDCD4–40S), 8XXM (state PDCD4–eIF3G–40S), 8XXN (state PDCD4–43S).
