# Supplementary material for: Human tumor suppressor PDCD4 directly interacts with ribosomes to repress translation
Source: Cell Res. 2024 Apr 19;34(7):522–5. doi: 10.1038/s41422-024-00962-z (PMC11217289; doi:10.1038/s41422-024-00962-z)
Supplement: Supplementary file 1 — Supplementary information, MATERIALS AND METHODS [file 41422_2024_962_MOESM1_ESM.docx]

**Supplementary Information**

**Human tumor suppressor PDCD4 directly interacts with ribosomes to repress translation**

Xianwen Ye^1,2^, Zixuan Huang^1,2^, Yi Li^1^, Mengjiao Wang^1^, Wanyu Meng^1^, Maojian Miao^1^, and Jingdong Cheng^1,*^

**MATERIALS AND METHODS**

**Gene cloning and cell culture**

The human *PDCD4* and *PYM1* genes were obtained from a cDNA library reverse transcribed from SK-HEP1 cells and cloned into a modified pcDNA5/FRT/TO plasmid (Invitrogen), resulting in the pcDNA5-PDCD4-StFLAG and pcDNA5-PYM1-StFLAG plasmids, each fused with a C-terminal 2xStrep and 3xFLAG tag. To generate mutant variants of PDCD4 (used for transient transfection of the HEK293T cells to detect their association with the 40S ribosome), site-directed mutagenesis was performed on the pcDNA5-PDCD4-StFLAG plasmid, resulting in the following mutants: 2A (W124A, Y131A), 3A (R102A, R103A, R110A), 5A (R102A, R103A, R110A, W124A, Y131A), Δ150-160, Δ100-160, M2A (D249A, D253A), C2A (D414A, D418A), and 4A (D249A, D253A, H358A, F359A). Additionally, the wild-type PDCD4 gene was inserted into the pLVX-EGFP-IRES-puro vector (Addgene, 128652), resulting in the pLVX-PDCD4-GFP plasmid (used to detect the cellular localization of PDCD4).

The generation of the stable PDCD4 and PYM1 overexpression HEK293/Flp-In/T-Rex cell lines (used for purification of the native PDCD4-ribosome complex) followed protocols adapted from previous works.^1,2^ Briefly, HEK 293/Flp-In/T-Rex cells (Invitrogen) were pre-cultured in a 10-cm dish for one day. After reaching 50% confluence, the cells were co-transfected with 0.5 μg of either pcDNA5-PDCD4-StFLAG or pcDNA5-PYM1-StFLAG plasmid together with 4.5 μg of recombinase plasmid pOG44 (Invitrogen). Following 48 hours of transfection, the cells were passaged and subjected to a 14-day selection with 200 μg/mL hygromycin B (Meilunbio, MB6158). Finally, validation of the selected cell lines was finally performed by Western blotting. Stable cell lines were cultured in high glucose (4.5 mg/mL) DMEM medium (Peiyuan, Shanghai) containing 10% FBS (Yeasen, Shanghai) and 1× penicillin/streptomycin.

Antibodies used in this study included anti-PDCD4 (12587-1-AP, Proteintech), anti-uS5 (A303-794A, Bethyl), anti-eL8 (2415S, CST), anti-eIF4A1 (A23514, Abclonal), anti-eIF3B (A9143, Abclonal), and anti-Flag (A8592, Sigma).

**Purification of native PDCD4-ribosome complexes**

Native PDCD4-associated complex was purified from the stable PDCD4-overexpressing or PYM1-overexpressing HEK293/Flp-In/T-Rex cell lines as previously described.^1,2^ Using PYM1- StFLAG overexpression cells as an example, to induce nutrient starvation, the stable cells were cultured for an additional 1 or 2 days beyond reaching full confluence. Briefly, after 4-5 days of culture, bait protein expression was induced with 1 µg/mL tetracycline for 24 hours. Prior to collection, cells were treated with 10 μg/mL cycloheximide (CHX) for 10 minutes. For each sample, a total of 50 dishes (15 cm) of cells were collected with a cell scraper, washed twice with cold 1× PBS buffer, and resuspended in lysis buffer (20 mM HEPES pH 7.4, 100 mM KOAc, 5 mM MgCl_2_, 1 mM DTT, 10 μg/mL CHX, 0.5 mM NaF, 1 mM Na_3_V_3_O_4_, 1× protease inhibitor mix). Cells were lysed by 10-15 strokes using a 15 mL Douncer homogenizer. The cell lysate was clarified by centrifugation at 10,000 × g for 15 minutes at 4 °C, and the supernatant was incubated with 200 μL Flag beads (A2220, Sigma) in a 50 mL centrifuge tube for 2 hours at 4 °C. After incubation, the beads were transferred to a small chromatography column, washed once with lysis buffer, and then washed three times with wash buffer (20 mM HEPES pH 7.4, 100 mM KOAc, 5 mM MgCl_2_, 10 μg/mL CHX). Finally, the protein complex was eluted with 500 μL elution buffer (20 mM HEPES pH 7.4, 100 mM KOAc, 5 mM MgCl_2_, 0.4 mg/mL 3× Flag peptide (P9801, beyotime)) for 45 minutes at 4 °C. The eluate was concentrated using a 100 kDa cut-off concentrator, and the final concentration was determined using a NanoDrop photometer. Protein quantification and identification were then performed by label-free protein quantification using LC-MS.

**Generation of PDCD4 knockout DLD-1 cells**

To generate PDCD4 knockout cells (used to detect the cellular localization of PDCD4), the parental DLD-1 cells were cultured in McCoy’s 5A medium (Peiyuan, Shanghai) supplemented with 10% FBS (Yeasen, Shanghai) and 1× penicillin/streptomycin. The sgRNA sequence (CCGGTGATGAAGAAAATGCT) designed to target the *PDCD4* gene was inserted into the pLentiCRISPR vector (AddGene, 52961), resulting in pLentiCRISPR-sgPDCD4. For lentiviral production, HEK293T cells were then pre-cultured in a six-well plate for one day. The 1 μg of pLentiCRISPR-sgPDCD4 plasmids were co-transfected into HEK293T cells together with the packaging plasmids 0.25 μg of pMD2.G (AddGene, 12259) and 0.75 μg of psPAX2 (AddGene, 12260). After 36-48 hours, the cell supernatant containing lentiviral particles was collected to infect DLD-1 cells with 10 μg/mL polybrene (Sigma, 107689). 24 hours after infection, the cells were passaged and subjected to a 2-day selection with 2 μg/mL puromycin (Meilunbio, MB2005). The selected cells were seeded in 96-well plates at a density of one cell per well by limited dilution. After culturing for 10-14 days, cell clones were picked and validated by Western blotting. The correct clone was expanded and cultured.

**Cellular localization of PDCD4**

The HEK293T cells were grown to 40-50% confluence in a 6-well plate. The cells were then co-transfected with 1 μg pLVX-PDCD4-GFP, 0.25 μg pMD2.G (addgene, #12259) and 0.75 μg psPAX2 (addgene, #12260) using PEI (YEASEN, 40816ES03). After 36-48 hours post-transfection, the supernatant was collected as virus for infecting target cells.

PDCD4 knockout DLD-1cells were seeded in 35-mm glass bottom dishes and infected with a sixfold diluted virus supplemented with 10 μg/mL polybrene (Sigma, 107689-10G) to approximate endogenous PDCD4 expression levels. 24 hours after infection, a portion of cells were treated as follows: 20 µM etoposide (csnpharm, 33419-42-0) for 3 hours, 125 µM cis-platinum (csnpharm, 15663-27-1) for 3 hours, 200 µM cytarabine (Ara-C, beyotime, 147-94-4) for 12 hours, glucose starvation for 4 hours, amino acid starvation for 4 hours, serum deprivation for 7 hours, and 100 nM Torin1 (csnpharm, 1222998-36-8) for 12 hours. Meanwhile, another portion of cells were washed once with PBS buffer and then incubated in the PBS buffer for different time points (0 min, 5 min, 10 min, 20 min and 40 min). After treatment, cells were fixed in 4% paraformaldehyde (Servicebio, G1101) in PBS for 15 minutes and washed three times with PBS for 5 minutes each. Cells were permeabilized with 0.4% Triton X-100 (Sangon Biotech, A110694-0100) in PBS for 8 minutes, washed three times, and blocked with 5% bovine serum albumin in PBS for 1 hour. After three washes, cells were stained with DAPI (Sigma, D9542) at 1:1000 in blocking buffer for 5 minutes and then washed three times. Finally, images were captured on a Leica TCS SP5 confocal microscope using 40x oil objectives.

**Distribution of endogenous PDCD4 protein over sucrose gradient**

To minimally stress the cells during the cell collection and lysis, a rapid cell lysis protocol was used. In detail, For the glucose-starved cells, HEK293T cells were plated in a 10 cm dish on the first day. The next day, when the cell reached 20-30% confluence, they were switched into glucose-deficient DMEM medium for 24 hours. For control cells, HEK293T cells were split on the second day and reached 20-30% confluence on the third day. After incubation on the third day, the cells were scratched down within the medium and then centrifuged at 500 × g for 30 seconds. Cell pellets were resuspended in 500 µL of hypotonic buffer (20 mM Tris-HCl pH 7.4, 10 mM NaCl, 2 mM MgCl_2_, 1 mM EDTA, 0.1% NP-40, 1× protease inhibitor mix) and lysed using an injection syringe equipped with a 0.45 mm × 16 mm needle for 1 stroke. The cell lysates were centrifuged at 500g for 30 seconds at 4 ℃, and the resulting supernatants were transferred to new microcentrifuge tubes and centrifuged again at 17,000g for 15 seconds at 4 ℃. Notably, no cycloheximide was used in this experiment. In general, we were able to complete the entire process in approximately 5 minutes. The supernatants were transferred to new microcentrifuge tubes. The samples were then fractionated on a 10-40% (w/v) sucrose gradient prepared in sucrose buffer solution (20 mM Tris-HCl pH 7.4, 10 mM NaCl, 2 mM MgCl_2_, 1 mM EDTA) using the Gradient Master (Biocomp). Following fractionation, the samples were centrifuged at 39,000 rpm for 4.5 hours at 4 °C using a SW41Ti rotor (Beckman). Protein fractions obtained from the sucrose gradient were precipitated with 10% trichloroacetic acid (TCA) overnight at 4 °C and then washed with acetone pre-chilled at -20 °C. The resulting pellets were resuspended in 40 µL of 1x SDS loading buffer for each fraction. The proteins were then separated by 10% SDS-PAGE, and the separated proteins were subjected to Western blot analysis.

**Immunoprecipitation**

HEK293T cells were plated in 10-cm dishes. The next day, when cells reaching 50% confluence, they were transfected separately with 4 μg pcDNA5-PDCD4-StFLAG or its mutants (2A, 3A, 5A, M2A, 4A, C2A) using PEI (YEASEN, 40816ES03). 48 hours after transfection, cells were washed twice in cold 1×PBS, and cell pellets were resuspended in 500 μL lysis buffer (20 mM HEPES pH 7.4, 100 mM KOAc, 5 mM MgCl_2_, 1 mM DTT, 0.5 mM NaF, 1 mM Na_3_V_3_O_4_, and 1× protease inhibitor mix). Cells were further lysed using an injection syringe equipped with a 0.45 mm × 16 mm needle. Cell lysates were clarified by centrifugation at 12,000 rpm for 20 minutes at 4 ℃. The cell supernatants were added to the new tubes and incubated with 12-15 μL Flag beads (A2220 Sigma) per sample at 4 ℃ for 2 hours. After incubation, the beads were washed three times with wash buffer (20 mM HEPES pH 7.4, 100 mM KOAc, 5 mM MgCl_2_). The proteins were eluted with 1× SDS loading buffer (50 mM Tris-HCl pH 6.8, 10% glycerol, 5% β-mercaptoethanol, 2% sodium dodecyl sulfate, 0.0005% bromophenol blue), and the resulting samples were separated on a 10% SDS-PAGE gel, and the interacting proteins were subjected to Western blot analysis.

**Sucrose gradient fractionation**

HEK293T cells were plated in 10-cm dishes. Upon reaching 50% confluence, cells were separately transfected with 4 μg of pcDNA5-PDCD4-StFLAG or its mutants (2A, 3A, 5A, Δ150-160, Δ100-160, M2A, C2A, 4A) using PEI (YEASEN, 40816ES03). After 36-48 hours after transfection, the cells were treated with 100 µg/mL cycloheximide (CHX, MCE, HY-12320) for 5 minutes to prevent ribosome runoff, followed by two washes with cold 1x PBS containing 100 µg/mL CHX. Cell pellets were then lysed with 500 µL of lysis buffer (20 mM HEPES pH 7.4, 100 mM KOAc, 5 mM MgCl_2_, 1 mM DTT, 100 μg/mL CHX, 0.5 mM NaF, 1 mM Na_3_V_3_O_4_, 1× protease inhibitor mix).

To disrupt the cells, the lysates were aspirated 3-5 times using 1mL syringes (with 0.45 mm × 16 mm needles). Subsequently, the cell lysates were centrifuged at 15,000 rpm for 15 minutes at 4 °C, and the resulting supernatants were carefully transferred to a new microcentrifuge tube. The samples were then subjected to fractionation on a 15-35% (w/v) sucrose gradient prepared in sucrose solution (20 mM HEPES pH 7.4, 100 mM KCl, 5 mM MgCl_2_, 1 mM DTT, 0.5 mM EDTA, 10 μg/mL CHX) using the Gradient Master (Biocomp). The prepared samples were centrifuged at 39,000 rpm for 4.5 hours at 4 °C using a SW41Ti rotor (Beckman). Protein fractions obtained from the sucrose gradient were precipitated with 10 % trichloroacetic acid (TCA) overnight at 4°C. Following precipitation, the samples were washed with acetone pre-chilled at -20°C. The resulting pellets were resuspended in 40 µL of 1x SDS loading buffer for each fraction. The proteins were then separated by 10% SDS-PAGE, and the separated proteins were subjected to Western blot analysis.

**Electron microscopy preparation and image processing**

Purified samples (PYM1-pullout sample and PDCD4-pullout sample, 3.5 μL each) were applied to precoated (2 nm carbon) R1.2/1.3 carbon-supported copper grids (Quantifoil), blotted for 4-5 s at 4°C, and plunge-frozen in liquid ethane using an FEI Vitrobot Mark IV. Data from the PYM1 sample were collected on a Titan Krios G4 cryo-electron microscope operating at 300 keV using EPU 2. The data were collected at a pixel size of 1.146 Å/pixel and within a defocus range of -1 to -2.5 μm using a Falcon IV direct electron detector under low dose conditions with a total dose of 50 e-/Å^2^. Meanwhile, data for the PDCD4 sample were collected on a Titan Krios G3 operating at 300 keV using serialEM. The data were collected with a pixel size of 1.064 Å/pixel and within a defocus range of -1 to -2.5 μm using a K3 direct electron detector under low dose conditions with a total dose of 58 e-/Å^2^. The original image stacks were dose-weighted, aligned, summed, and drift-corrected using MotionCor2.^3^ Contrast transfer function (CTF) parameters and resolutions were estimated for each micrograph using GCTF.^4^ Micrographs with an estimated resolution of less than 5 Å and astigmatism of less than 5% were manually inspected for contamination or carbon breakage.

A total of 12,457 good micrographs were selected for the PDCD4-pullout sample. After automated particle picking using Gautomatch (https://www2.mrc-lmb.cam.ac.uk/download/gautomatch-053/), a total number of 2,878,720 particles were extracted and then subjected to 2D classification in cryoSPARC,^5^ resulting in 1,657,887 particles representing the 40S ribosomal subunit. These particles were then imported into Relion^6^ for extensive 3D classification. After several rounds of (focused) classification, three distinct classes showing PDCD4 density were selected and named PDCD4-40S, PDCD4-eIF3G, and PDCD4-43S states. Subsequent 3D refinement and multi-body refinement were performed in Relion^6^ to obtain the final reconstructions. The detailed sorting scheme is illustrated in Supplementary Information Fig. S2.

Similarly, a total of 13,347 good micrographs were selected for the PYM1-pullout sample. Automatic particle picking was performed in Gautomatch (https://www2.mrc-lmb.cam.ac.uk/download/gautomatch-053/) without the use of a reference. Picked particles (1,906,164 particles) were extracted in Relion 3.1 and then subjected to 2D classification in cryoSPARC.^5^ Following this, 670,975 good particles that clearly represented the 40S ribosomal subunit were selected for 3D classification in Relion 3.1.^6^ The first round of 3D classification separated four different 40S classes. The class representing the 43S PIC was selected and subjected to further rounds of classification, resulting in the identification of the 43S state III. Focused classification using a soft mask covering the mRNA entry channel region on the remaining particles resulted in two additional states: the PDCD4-43S state and the 43S state II. Since the two states of 43S PIC (state II and III) have already been discussed before,^7^ we focused only on the PDCD4-43S state. For this, 3D refinement and multi-body refinement were performed in Relion^6^ to obtain the final maps. To improve the reconstruction of the C-terminal PDCD4 and eIF4A region, we generated a soft mask specifically targeting this area. Subsequently, we performed a focused refinement using this mask to improve the local resolution of this area. The detailed sorting scheme is presented in Supplementary Information Fig. S3.

**Model building and refinement**

We used the human 43S PIC structures (PDB: 7A09, 6ZVJ)^7^ as templates for rigid body fitting into the cryo-EM maps. Manual adjustments were made in Coot,^8^ especially to the 40S head. The RBR of PDCD4 was built manually in Coot.^8^ For the PDCD4-43S state, the crystal structures of the PDCD4-eIF4A (PDB: 3EIQ, 2ZU6)^9,10^ were used for rigid body fitting in the density map near the mRNA entry site. The structure of the eIF3G and PDCD4 complex in the states PDCD4-eIF3G-40S and PDCD4-43S was adapted from an AlphaFold-predicted structure using the AlphaFold multimer.^11^ All the side chains of the C-terminus of PDCD4 and eIF4A in the PDCD4-43S state are removed from the final models due to their low local resolution.

The final models were real-space refined with secondary structure restraints using the PHENIX suite.^12^ Final model evaluation was performed using MolProbity.^13^ Maps were filtered according to their local resolution estimation using both Relion^6^and DeepEMhancer.^14^ Maps and models were visualized and figures were generated using ChimeraX.^15^

**ADDITIONAL REFERENCES**

1. Ameismeier, M., Cheng, J., Berninghausen, O. & Beckmann, R. *Nature* **558**, 249-253 (2018).

2. Wyler, E. *et al.* *RNA* **17**, 189-200 (2011).

3. Zheng, S. Q. *et al.* *Nat Methods* **14**, 331-332 (2017).

4. Zhang, K. *Journal of structural biology* **193**, 1-12 (2016).

5. Punjani, A., Rubinstein, J. L., Fleet, D. J. & Brubaker, M. A. *Nat Methods* **14**, 290-296 (2017).

6. Zivanov, J. *et al.* *eLife* **7**, e42166 (2018).

7. Kratzat, H. *et al.* *The EMBO journal* **40**, e105179 (2021).

8. Emsley, P. & Cowtan, K. *Section D, Biological crystallography* **60**, 2126-2132 (2004).

9. Loh, P. G. *et al.* *The EMBO journal* **28**, 274-285 (2009).

10. Chang, J. H. *et al.* *Proceedings of the National Academy of Sciences of the United States of America* **106**, 3148-3153 (2009).

11. Jumper, J. *et al.* *Nature* **596**, 583-589 (2021).

12. Adams, P. D. *et al.* *Acta crystallographica. Section D, Biological crystallography* **66**, 213-221 (2010).

13. Chen, V. B. *et al.* *Acta crystallographica. Section D, Biological crystallography* **66**, 12-21 (2010).

14. Sanchez-Garcia, R. *et al.* *Commun Biol* **4**, 874 (2021).

15. Goddard, T. D. *et al.* *Protein Sci* **27**, 14-25 (2018).
