# Supplementary material for: Human tumor suppressor PDCD4 directly interacts with ribosomes to repress translation
Source: Cell Res. 2024 Apr 19;34(7):522–5. doi: 10.1038/s41422-024-00962-z (PMC11217289; doi:10.1038/s41422-024-00962-z)
Supplement: Supplementary file 2 — Supplementary information, Fig. S1 [file 41422_2024_962_MOESM2_ESM.pdf]

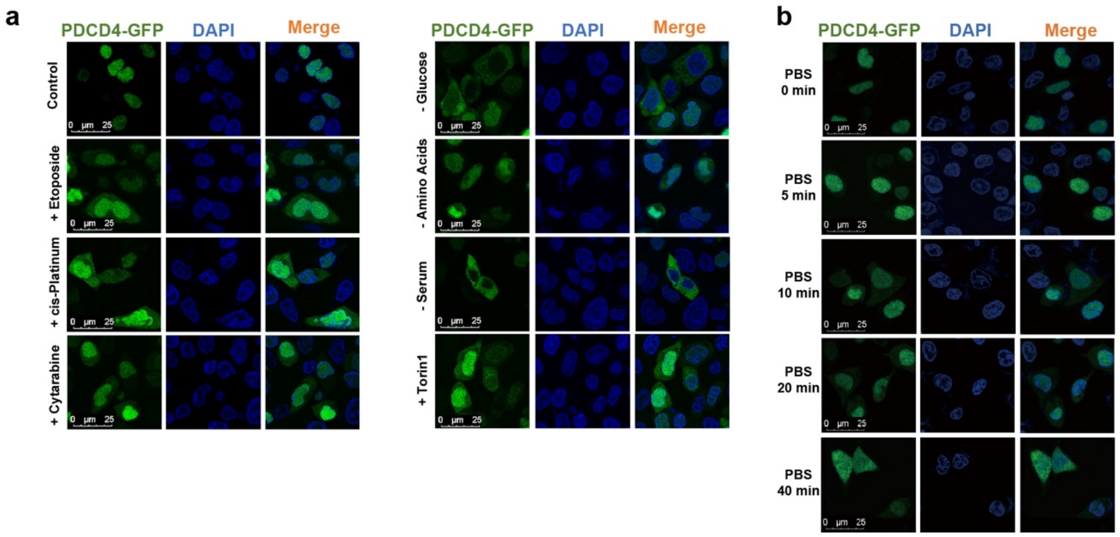

Supplementary information, Fig. S1 The cellular localization of PDCD4. a, b The intracellular localization of PDCD4 is affected by DNA damage and nutrient starvation. The PDCD4-GFP fusion protein was expressed in PDCD4 knockout DLD-1 cells at the endogenous level. After 24 hours, cells were treated with different stimuli (**a**): 20 μM etoposide for 3 hours, 125 μM cis-platinum for 3 hours, 200 μM cytarabine (Ara-C) for 12 hours, glucose starvation for 4 hours, amino acid starvation for 4 hours, serum deprivation for 7 hours, and 100 nM Torin1 for 12 hours; or incubated with PBS buffer (**b**)for different time points (0 min, 5 min, 10 min, 20 min, and 40 min). Notably, a small amount of PDCD4 had translocated to the cytoplasm after only 10 minutes of incubation in buffer, whereas after 40 minutes it was evenly distributed between the nucleus and the cytosol. Merged images are shown, with the blue signals representing the nuclei stained with DAPI and the green GFP signals indicating the localization of the PDCD4-GFP fusion protein.
