# Supplementary material for: Human tumor suppressor PDCD4 directly interacts with ribosomes to repress translation
Source: Cell Res. 2024 Apr 19;34(7):522–5. doi: 10.1038/s41422-024-00962-z (PMC11217289; doi:10.1038/s41422-024-00962-z)
Supplement: Supplementary file 3 — Supplementary information, Fig. S2 [file 41422_2024_962_MOESM3_ESM.pdf]

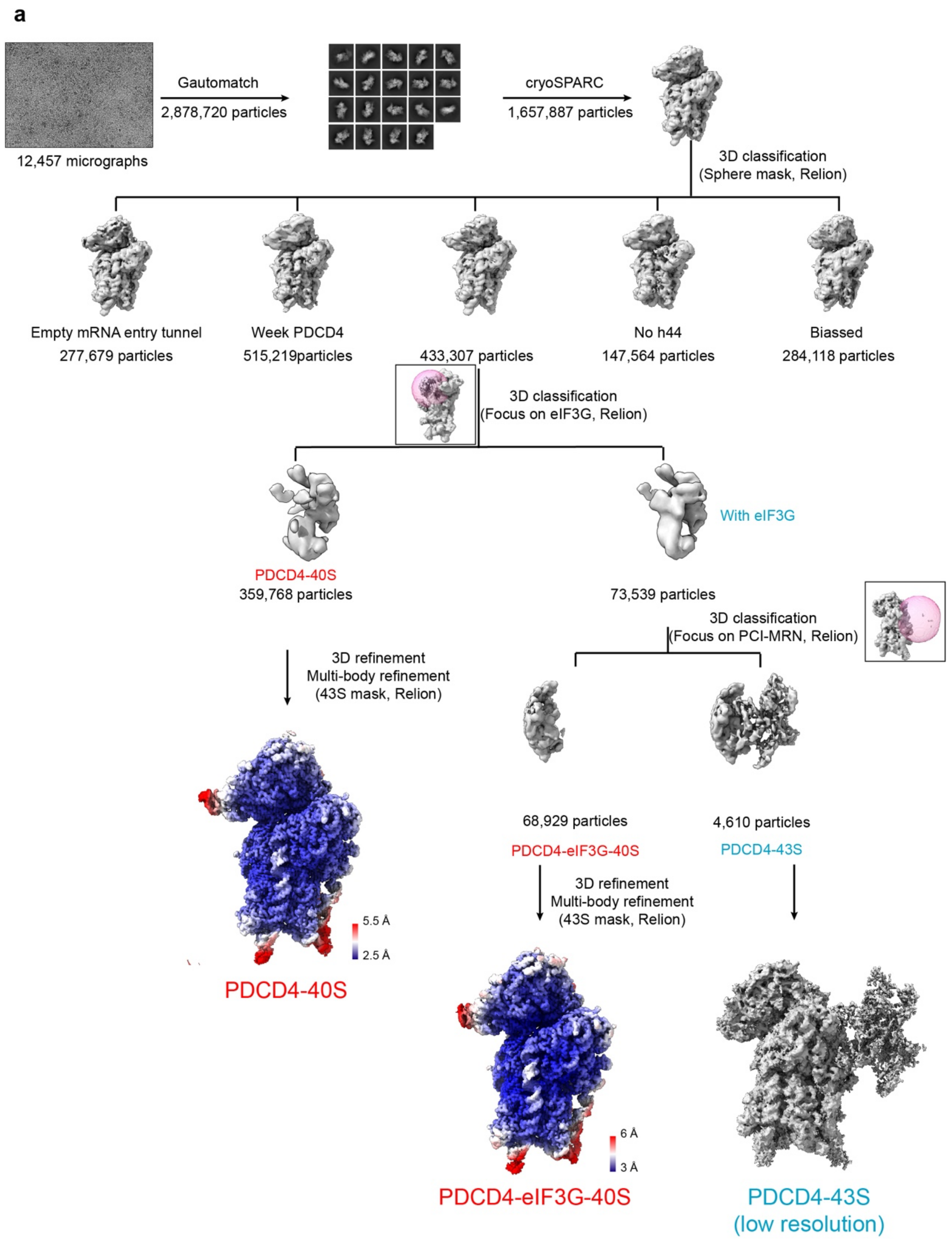

Supplementary information, Fig. S2 The sorting scheme of the PDCD4 cryo-EM dataset. **a** The sorting scheme of the cryo-EM dataset from the PDCD4 sample. The masks and software used during data processing are shown on the side. Finally, the final two maps are colored according to their local resolution distribution, while the PDCD4-43S map is colored in gray.
