# Supplementary material for: Human tumor suppressor PDCD4 directly interacts with ribosomes to repress translation
Source: Cell Res. 2024 Apr 19;34(7):522–5. doi: 10.1038/s41422-024-00962-z (PMC11217289; doi:10.1038/s41422-024-00962-z)
Supplement: Supplementary file 4 — Supplementary information, Fig. S3 [file 41422_2024_962_MOESM4_ESM.pdf]

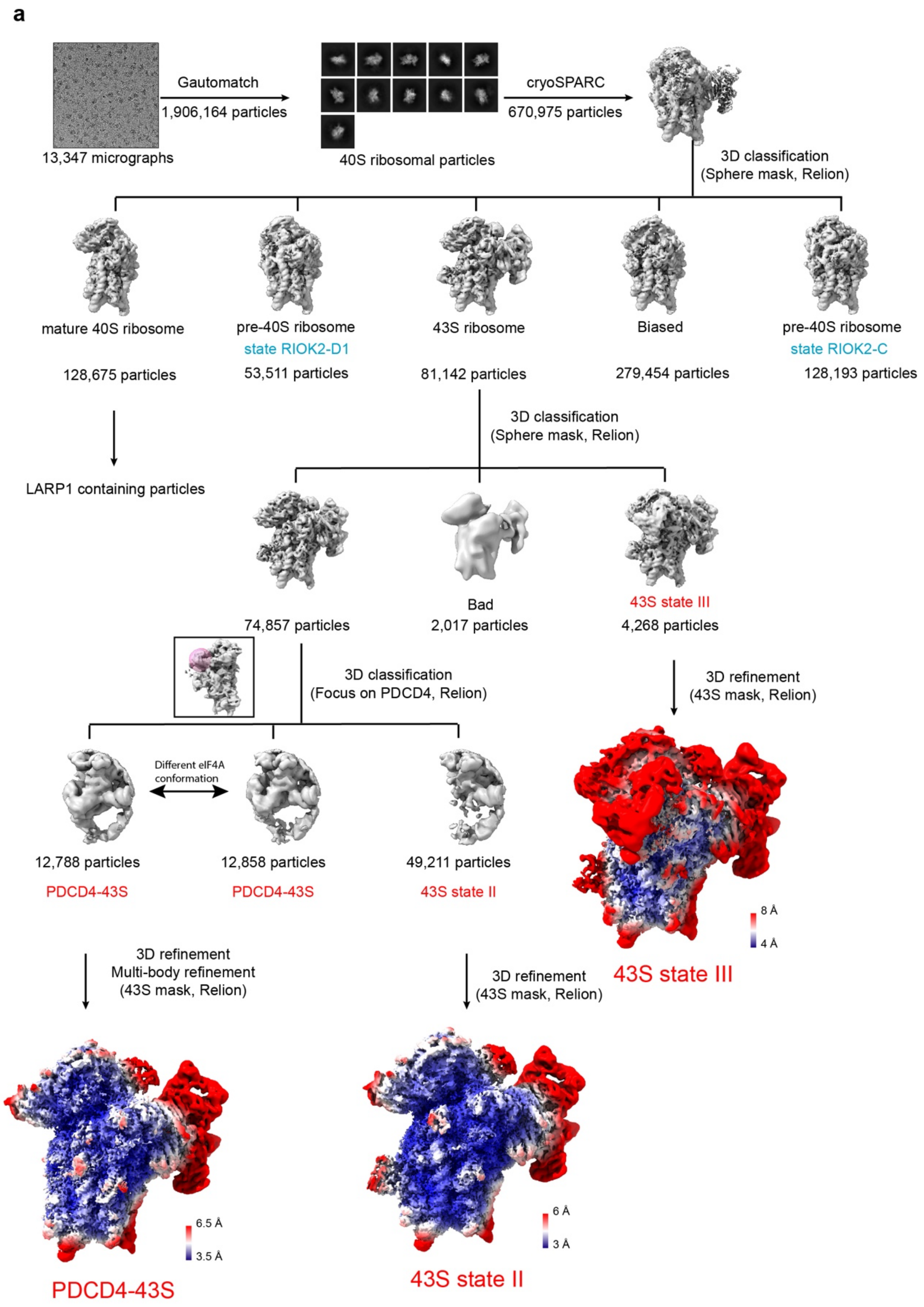

Supplementary information, Fig. S3 The sorting scheme of the PYM1 cryo-EM dataset. a The sorting scheme of the cryo-EM dataset from the PYM1 sample. The masks and software used during data processing are shown on the side. In the end, three classes were selected and colored according to their local resolution. Only the PDCD4-43S state has PDCD4 bound. Notably, 43S "state II" and "state III" are normal 43S PIC assembly intermediates.
