# Supplementary material for: Human tumor suppressor PDCD4 directly interacts with ribosomes to repress translation
Source: Cell Res. 2024 Apr 19;34(7):522–5. doi: 10.1038/s41422-024-00962-z (PMC11217289; doi:10.1038/s41422-024-00962-z)
Supplement: Supplementary file 5 — Supplementary information, Fig. S4 [file 41422_2024_962_MOESM5_ESM.pdf]

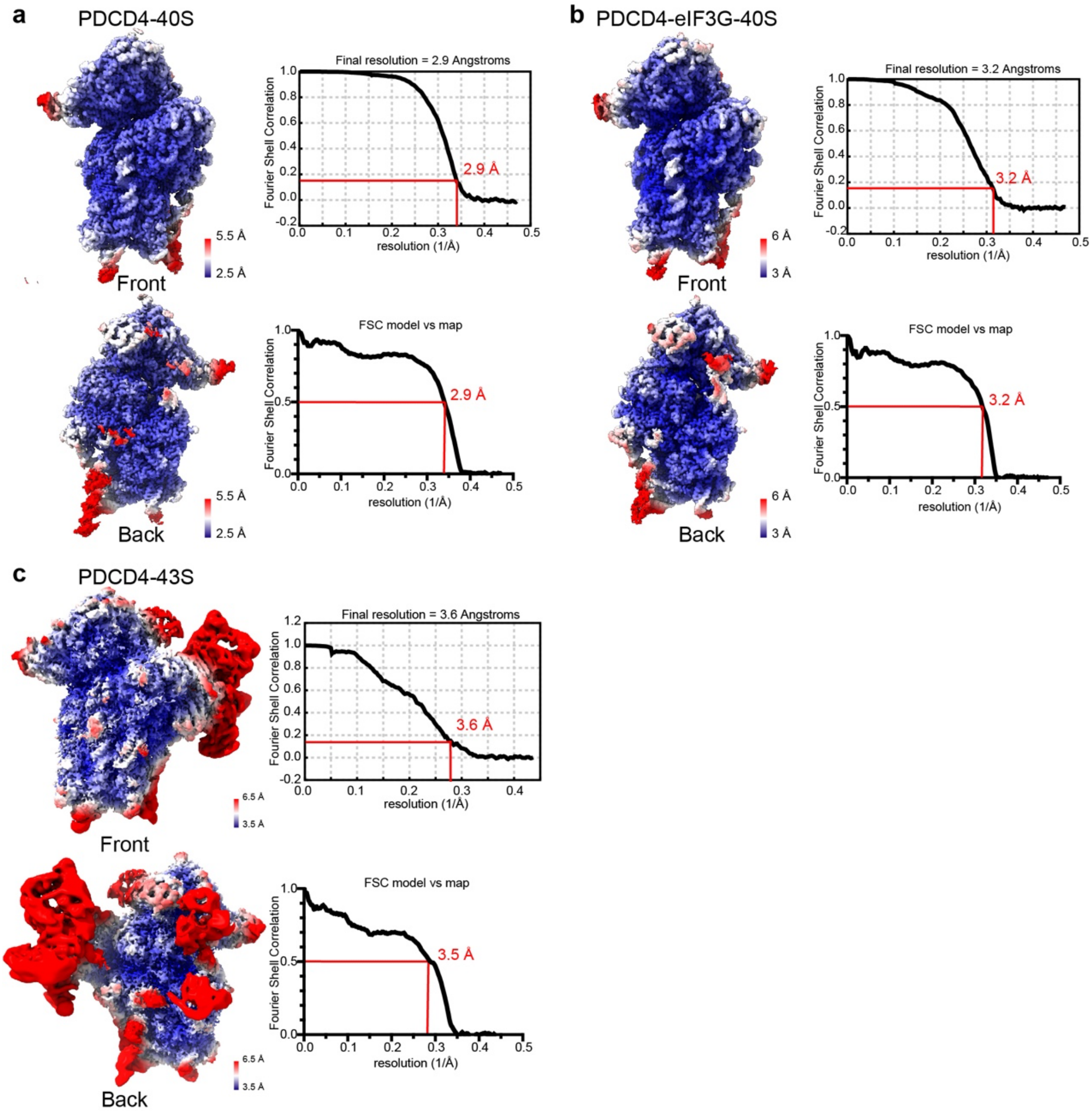

Supplementary information, Fig. S4 The local resolution and FSC curves of the PDCD4-40S ribosome structures. a-c Two different views (front and back) of the cryo-EM maps of the PDCD4-40S (**a**), PDCD4-eIF3G-40S (**b**), and PDCD4-43S (**c**) states are colored according to their local resolution estimation (left). The corresponding Fourier shell correlation (FSC) curves (top) of the maps and the model-to-map correlation curves (bottom) were calculated in Relion. The corresponding resolution was estimated using either the 0.143 or 0.5 cutoff criterion (red line).
