# Supplementary material for: Human tumor suppressor PDCD4 directly interacts with ribosomes to repress translation
Source: Cell Res. 2024 Apr 19;34(7):522–5. doi: 10.1038/s41422-024-00962-z (PMC11217289; doi:10.1038/s41422-024-00962-z)
Supplement: Supplementary file 6 — Supplementary information, Fig. S5 [file 41422_2024_962_MOESM6_ESM.pdf]

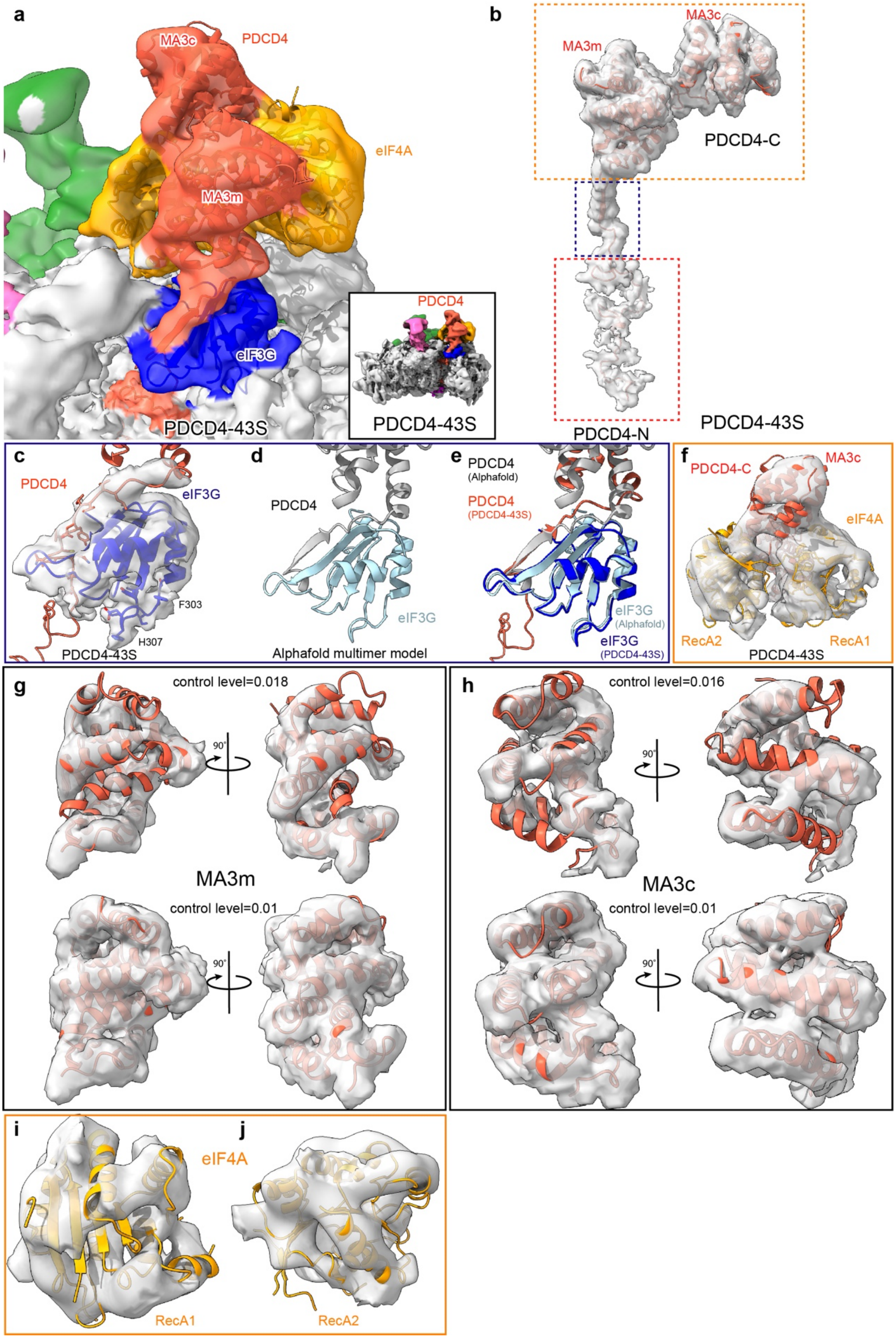

Supplementary information, Fig. S5 Cryo-EM structures of PDCD4, eIF3G and eIF4A.
a Zoomed-in view of the C-terminal region of PDCD4 in the PDCD4-43S state. The position of this region within the context of the 40S ribosome is depicted as an insert. PDCD4 is shown

in red, eIF3G in blue, and eIF4A in orange, all of which are surrounded by transparent density maps. **b** Isolated density of PDCD4 along with the molecular model to illustrate the rigid body fit. The density map is derived from the focused refined map masking the C-terminal region of PDCD4 and eIF4A. **c** Close-up view displaying the rigid body fit of eIF3G and the eIF3G binding region of PDCD4. The eIF3G binding region of PDCD4 and its binding partner eIF3G are shown as ribbons. **d** Molecular model of the Alphafold-predicted eIF3G (light blue) and PDCD4 (gray) complex structure. **e** Superimposition of the Alphafold-predicted eIF3G (light blue) and PDCD4 (gray) complex with the solved eIF3G (blue) and PDCD4 (red) complex structure in the PDCD4-43S state. **f** The C-terminal MA3 domains have sufficient resolution to fit all secondary structures. Moreover, the density of eIF4A (orange) is sufficient to fit the RecA domain structure. **g, h** Two different views of the MA3m (**g**) and MA3c (**h**) domains of PDCD4 illustrating their fitting within the density maps. The density maps are shown at two different contour levels. **i, j** Fitting of the RecA1 (**i**) and RecA2 (**j**) domains of eIF4A.
