# Supplementary material for: Human tumor suppressor PDCD4 directly interacts with ribosomes to repress translation
Source: Cell Res. 2024 Apr 19;34(7):522–5. doi: 10.1038/s41422-024-00962-z (PMC11217289; doi:10.1038/s41422-024-00962-z)
Supplement: Supplementary file 7 — Supplementary information, Fig. S6 [file 41422_2024_962_MOESM7_ESM.pdf]

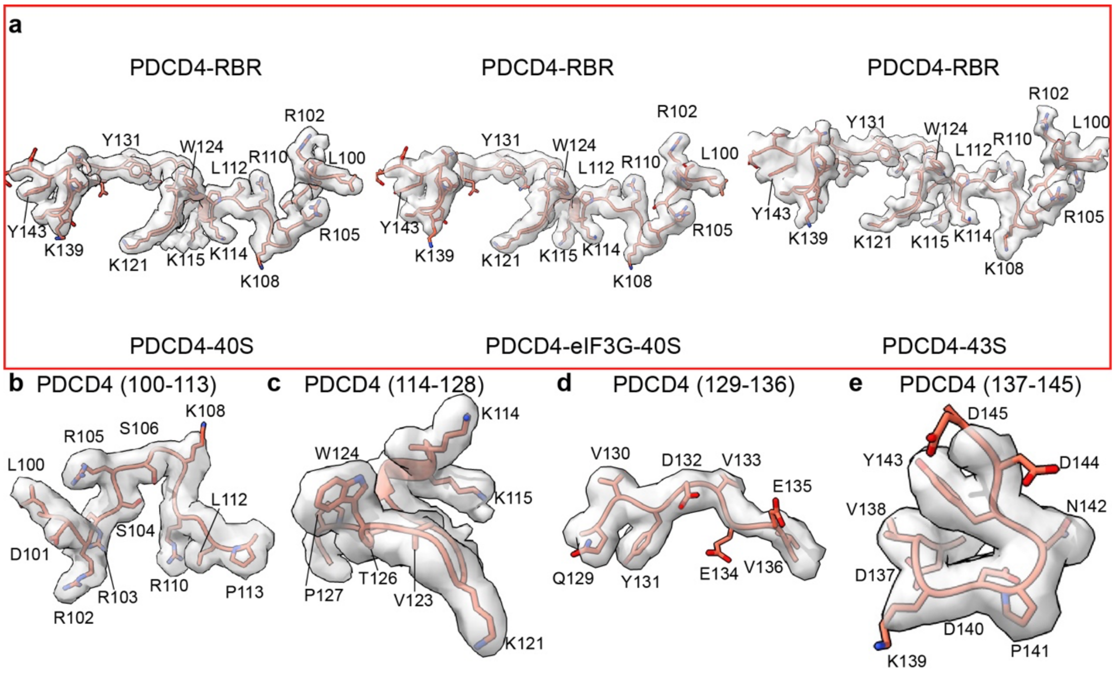

Supplementary information, Fig. S6 Cryo-EM structures of the PDCD4-RBR. a Cryo-EM structures of the PDCD4-RBR region in the PDCD4-40S (left), PDCD4-eIF3G-40S (middle) and PDCD4-43S (right) states. All the models are shown as sticks surrounded by transparent density maps. **b-e** The isolated density maps of the PDCD4-40S state for the following regions: aa. 100-113 (**b**), aa. 114-128 (**c**), aa. 129-136 (**d**) and aa. 137-145 (**e**). All the residues (except Ala and Gly) are labeled.
