# Supplementary material for: Human tumor suppressor PDCD4 directly interacts with ribosomes to repress translation
Source: Cell Res. 2024 Apr 19;34(7):522–5. doi: 10.1038/s41422-024-00962-z (PMC11217289; doi:10.1038/s41422-024-00962-z)
Supplement: Supplementary file 8 — Supplementary information, Fig. S7 [file 41422_2024_962_MOESM8_ESM.pdf]

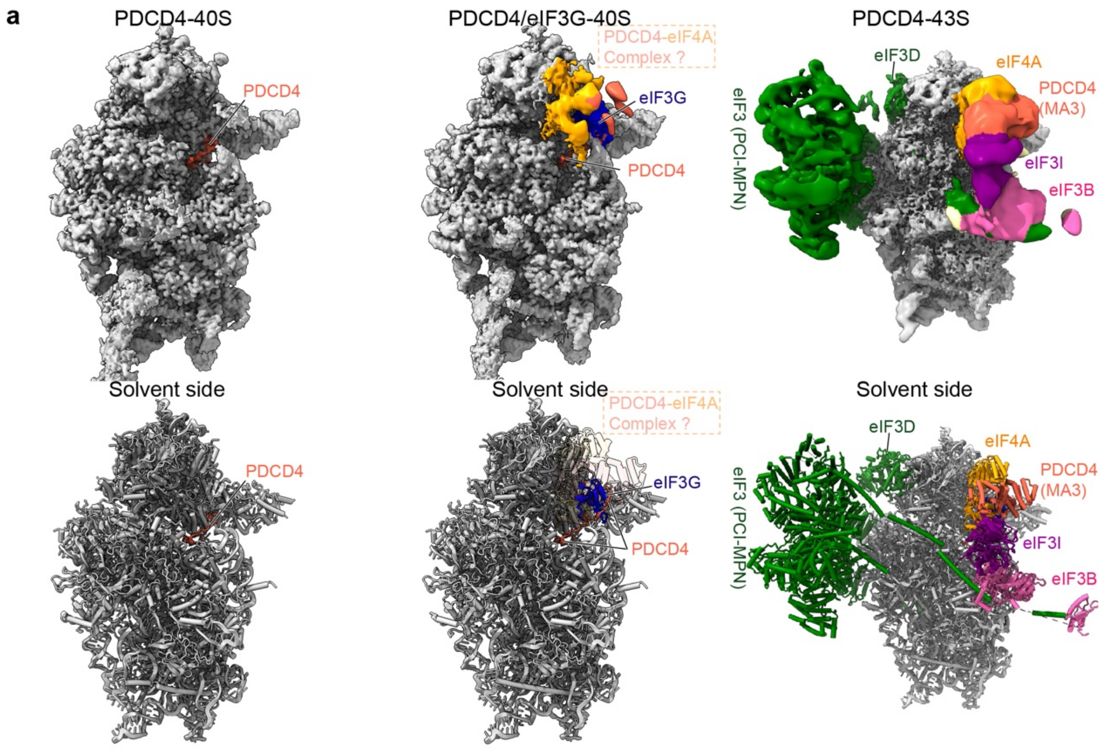

Supplementary information, Fig. S7 Backview of the PDCD4-ribosome structures. a Backviews of the cryo-EM structures of PDCD4-40S (left), PDCD4-eIF3G-40S (middle), and PDCD4-43S (right). The cryo-EM density maps are shown in the top row, while the built molecular models are shown in the bottom row. The 40S ribosome is colored gray, while PDCD4 (red) and initiation factors are colored in different colors (eIF4A: orange; eIF3G: blue; eIF1: magenta; eIF3I: purple; the PCI-MPN module: green; eIF3D: green). The cryo-EM maps are derived from the multibody refined maps and local resolution filtered by either Relion or DeepEMhancer. The density of the PDCD4-eIF4A complex in the PDCD4-eIF3G-40S state is shown at different contour levels, while the molecular model for the PDCD4-eIF4A complex in this state is shown transparently, indicating that it serves only as a placeholder.
