# Supplementary material for: Human tumor suppressor PDCD4 directly interacts with ribosomes to repress translation
Source: Cell Res. 2024 Apr 19;34(7):522–5. doi: 10.1038/s41422-024-00962-z (PMC11217289; doi:10.1038/s41422-024-00962-z)
Supplement: Supplementary file 9 — Supplementary information, Fig. S8 [file 41422_2024_962_MOESM9_ESM.pdf]

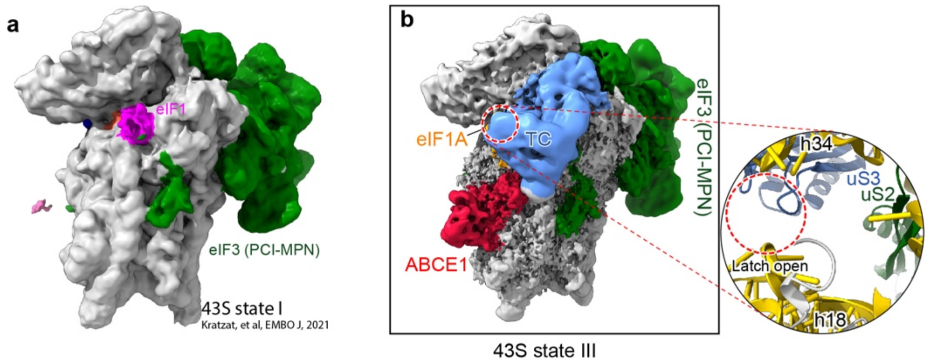

Supplementary information, Fig. S8 The PDCD4-43S state closely resembles the published 43S "State I". a Front view of the published 43S "State I" map. The map is colored according to our PDCD4-43S molecular model. **b** Cryo-EM map of the 43S PIC "State III" (PDB:7A09) filtered according to its local resolution. The zoomed-in image on the right highlights the "latch" region (red circle) within the mRNA channel. Note that compared with the closed latch in PDCD4-43S, it is opened to accommodate incoming mRNA in the 43S PIC "State III".
