# Supplementary material for: Human tumor suppressor PDCD4 directly interacts with ribosomes to repress translation
Source: Cell Res. 2024 Apr 19;34(7):522–5. doi: 10.1038/s41422-024-00962-z (PMC11217289; doi:10.1038/s41422-024-00962-z)
Supplement: Supplementary file 10 — Supplementary information, Fig. S9 [file 41422_2024_962_MOESM10_ESM.pdf]

a

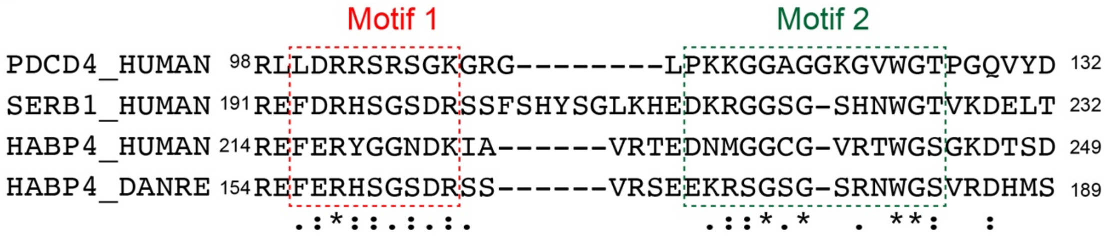

Supplementary information, Fig. S9 Sequence alignment of the conserved motifs of RBR. **a** Sequence alignment of the RBRs of PDCD4, SERBP1 and HABP4 showing that motifs 1 (indicated by the red dotted box) and 2 (indicated by the green dotted box) are conserved. Conservation is indicated by the dots and stars at the bottom.
