# Supplementary material for: Human tumor suppressor PDCD4 directly interacts with ribosomes to repress translation
Source: Cell Res. 2024 Apr 19;34(7):522–5. doi: 10.1038/s41422-024-00962-z (PMC11217289; doi:10.1038/s41422-024-00962-z)
Supplement: Supplementary file 11 — Supplementary information, Fig. S10 [file 41422_2024_962_MOESM11_ESM.pdf]

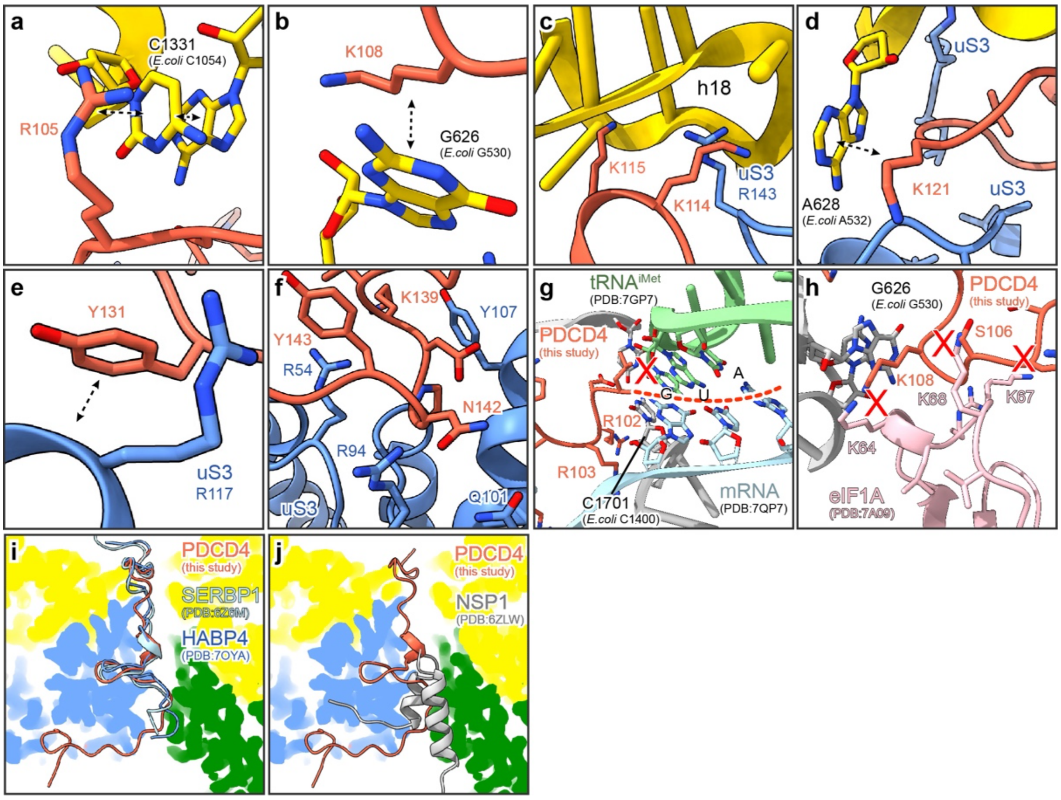

Supplementary information, Fig. S10 The interactome of PDCD4-RBR. a-f Detailed interactions between RBR and the 40S subunit: R105 stacks with base C1331 (**a**); K108 stacks with base G626 (**b**); K114/K115 interact with h18 of 18S rRNA (**c**); K121 stacks with base A628 (**d**); Y131 stacks with the backbone of residue R117 of uS3 (**e**); A region (aa. 139-143) of PDCD4 inserts into a surface pocket in uS3 (**f**). Key residues and nucleobases are indicated in all panels. Stacking interactions are indicated by black bidirectional arrows. **g** Structural comparison of the PDCD3-43S state with the 48S IC (PDB: 7GP7) showing the direct clash between PDCD4 (red) and the AUG start codon (light blue) and the cognate tRNA (light green). The putative position of the missing N-terminus of PDCD4 is shown as a dashed line. **h** Structural comparison of the PDCD4-43S state with the 43S PIC "State III" (PDB:7A09) showing the direct clash between PDCD4 (red) and eIF1A (pink). The direct clash sites are indicated by red crosses. **i** Overlay of PDCD4 (red), SERBP1 (light blue, PDB: 6Z6M) and HABP4 (blue, PDB: 7OYA) showing that all three adopt a similar conformation within the mRNA entry tunnel. **j** Structural comparison showing that PDCD4 (red) binds to a position similar to that of the NSP1 protein (gray, PDB: 6ZLW) of SARS-CoV-2 in the 40S ribosome.
