# Supplementary material for: Human tumor suppressor PDCD4 directly interacts with ribosomes to repress translation
Source: Cell Res. 2024 Apr 19;34(7):522–5. doi: 10.1038/s41422-024-00962-z (PMC11217289; doi:10.1038/s41422-024-00962-z)
Supplement: Supplementary file 12 — Supplementary information, Fig. S11 [file 41422_2024_962_MOESM12_ESM.pdf]

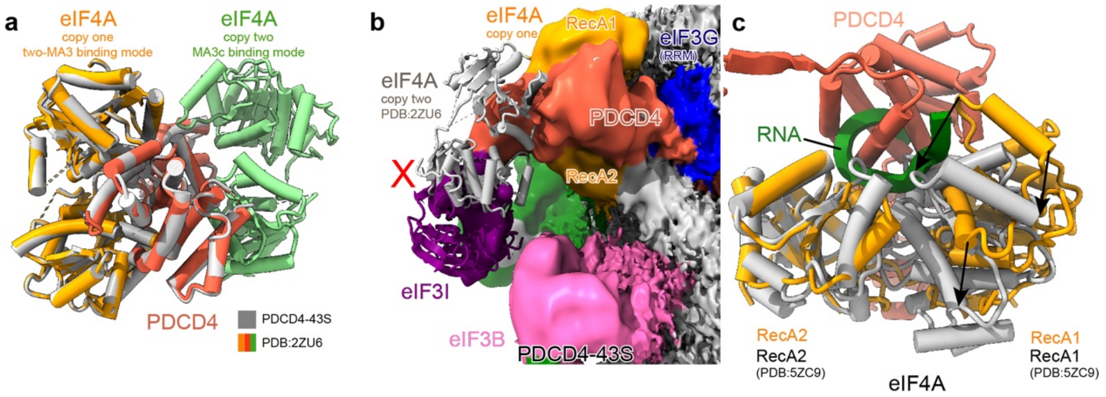


**Supplementary information, Fig. S11 Comparative structural analysis of the PDCD4-eIF4A complex. a** Superimposition of the PDCD4-eIF4A complex as found in the PDCD4-43S complex (gray) with the crystal structure (PDB: 2ZU6) showing that they have similar conformations except for the presence of an extra copy of eIF4A in the crystal structure. **b** Superimposition of the crystal structure of PDCD4-eIF4A (PDB: 2ZU6) on the PDCD4-43S state aligned based on PDCD4. The direct clash between the second copy of eIF4A (gray) in the crystal structure and eIF3I is indicated by a red cross. RRM: RNA recognition motif. **c** The superimposition of the PDCD4-eIF4A complex in this study with the crystal structure of the eIF4A complex with mRNA (PDB: 5ZC9). PDCD4 spatially clashes with the mRNA. Moreover, the two RecA domains in the PDCD4-eIF4A complex are in an open conformation and are not able to bind to mRNA. The RecA1 domain must move downward to adopt the active conformation, as indicated by the black arrow.
