# Supplementary material for: Human tumor suppressor PDCD4 directly interacts with ribosomes to repress translation
Source: Cell Res. 2024 Apr 19;34(7):522–5. doi: 10.1038/s41422-024-00962-z (PMC11217289; doi:10.1038/s41422-024-00962-z)
Supplement: Supplementary file 13 — Supplementary information, Fig. S12 [file 41422_2024_962_MOESM13_ESM.pdf]

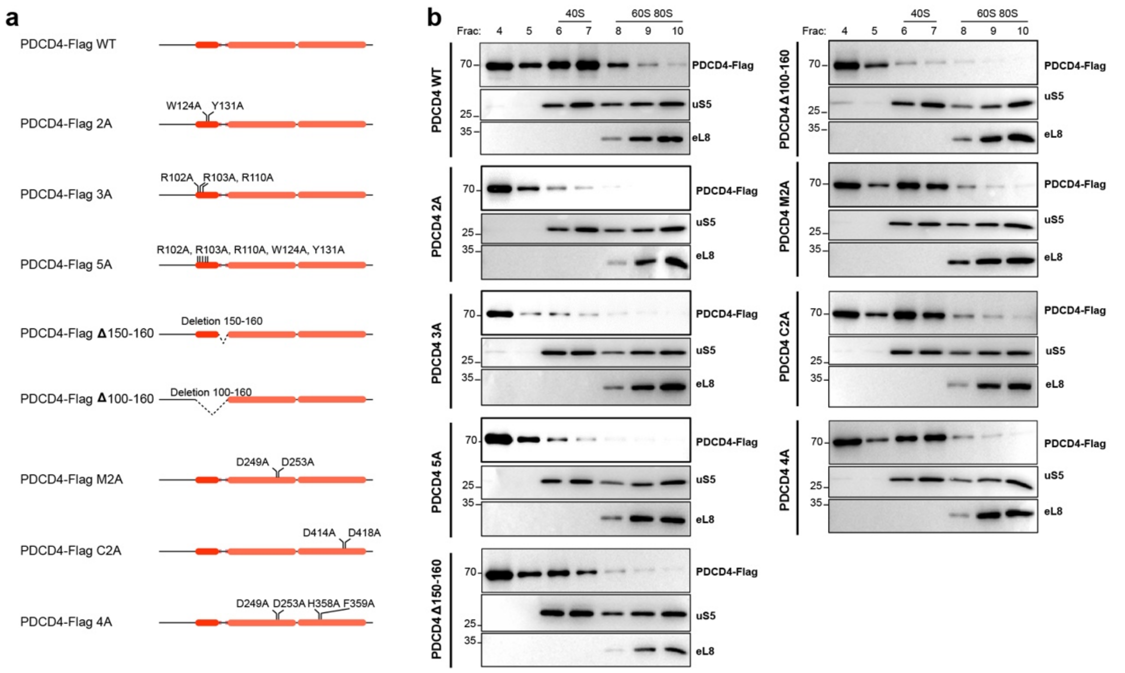

Supplementary information, Fig. S12 PDCD4 associates with ribosomes independent of its interaction with eIF4A. a Schematic showing the mutated residues of the PDCD4 protein in different variants. This included the following mutations: “3A” (R102A, R103A, and R110A) targeting “Motif 1”, “2A” (W124A and Y131A) targeting “Motif 2”, “5A” (a combination of “2A” and “3A”), deletion of amino acids 150-160 (Δ150-160) to disrupt the eIF3G interaction, deletion of amino acids 100-160 (Δ100-160) removing the RBR and eIF3G interacting region, “M2A” (D249A and D253A) in the MA3m domain only affecting the “two-MA3 binding mode” interface, “C2A” (D414A and D418A) in the MA3c domain only affecting the “MA3c binding mode” interface, and “4A” (D249A, D253A, H358A, and F359A) in the “two-MA3 binding mode” interface. **b** Cytoplasmic extracts isolated from human HEK293T cells transiently transfected with different PDCD4-Flag (WT) and its mutants (2A, 3A, 5A, Δ150-160, Δ100-160, M2A, C2A and 4A) were analyzed by sucrose density gradient (15-35%) centrifugation. Cycloheximide was used to prevent ribosome run-off. The fractions were precipitated with 10% trichloroacetic acid and separated by 10% SDS-PAGE. The distribution of PDCD4 mutants was revealed by Western blotting using anti-Flag antibodies. The position of the different ribosome populations is indicated by Western blotting using antibodies against uS5 (representing the 40S ribosome) and eL8 (representing the 60S ribosome). Fractions containing predominantly 40S subunits, 60S subunits and 80S ribosomes are marked.
