# Supplementary material for: Human tumor suppressor PDCD4 directly interacts with ribosomes to repress translation
Source: Cell Res. 2024 Apr 19;34(7):522–5. doi: 10.1038/s41422-024-00962-z (PMC11217289; doi:10.1038/s41422-024-00962-z)
Supplement: Supplementary file 14 — Supplementary information, Table S1 [file 41422_2024_962_MOESM14_ESM.pdf]

Supplementary information, Table S1 Cryo-EM data collection, refinement and validation statistics

	PDCD4-40S	PDCD4-eIF3G-40S	PDCD4-43S
**Data collection and processing**			
Magnification	81,000	81,000	105,000
Voltage (kV)	300	300	300
Electron exposure (e–/Å^2)	58	58	50
Defocus range (μm)	-1 to -2.5	-1 to -2.5	-1 to -2.5
Pixel size (Å)	1.064	1.064	1.146
Symmetry imposed	*C1*	*C1*	*C1*
Initial particle images (no.)	2,878,720	2,878,720	1,906,164
Final particle images (no.)	359,768	68,929	12,788
Map resolution (Å)	2.9	3.2	3.6
FSC threshold	0.143	0.143	0.143
**Refinement**			
Initial model used (PDB code)		6ZVJ/7A09	
Model resolution (Å)	2.9	3.2	3.5
FSC threshold	0.5	0.5	0.5
Map sharpening *B* factor (Å^2)	-94	-86	-78
Model composition			
Non-hydrogen atoms	76,240	76,934	114,615
Protein residues	4,975	5,703	10,313
RNA	1,723	1,723	1,723
Ligands	3	3	3
B factors (Å^2)	29.21	40.02	40.52
Protein	28.39	38.30	44.50
RNA	30.10	41.94	32.02
Ligand	17.52	20.70	22.51
R.m.s. deviations			
Bond lengths (Å)	0.005	0.005	0.006
Bond angles (°)	0.824	0.886	1.035
Validation			
MolProbity score	1.75	1.81	1.87
Clashscore	5.82	6.57	7.25
Poor rotamers (%)	0.58	0.02	0.17
Ramachandran plot			
Favored (%)	93.39	93.09	92.70
Allowed (%)	6.16	6.67	7.16
Disallowed (%)	0.45	0.23	0.14
**EMDB**	38752	38753	38754
**PDB**	8XXL	8XXM	8XXN
