# Supplementary material for: Human tumor suppressor PDCD4 directly interacts with ribosomes to repress translation
Source: Cell Res. 2024 Apr 19;34(7):522–5. doi: 10.1038/s41422-024-00962-z (PMC11217289; doi:10.1038/s41422-024-00962-z)
Supplement: Supplementary file 15 — Supplementary information, Table S2 [file 41422_2024_962_MOESM15_ESM.pdf]

Supplementary information, Table S2 Validation statistics for PDCD4 protein only

	**PDCD4 protein in (PDCD4-40S)**	**PDCD4 protein in (PDCD4-eIF3G-40S)**	**PDCD4 protein in (PDCD4-43S)**
Model composition			
Non-hydrogen atoms	346	372	1,841
Protein residues	46	49	347
B factors (Å^2)			
Protein	24.97	30.98	55.37
R.m.s. deviations			
Bond lengths (Å)	0.0049	0.0052	0.0063
Bond angles (°)	1.12	1.11	1.26
Validation			
MolProbity score	1.61	1.49	1.13
Clashscore	2.94	1.38	1.06
Poor rotamers (%)	0	0	0
Ramachandran plot			
Favored (%)	90.91	87.23	95.34
Allowed (%)	9.09	12.77	4.66
Disallowed (%)	0	0	0
CC (model vs map)	0.779	0.750	0.483
